# The CASPER preparation program innovation: increasing self-perceived competence and confidence of underrepresented applicants on the novel CASPER Snapshot and CanMEDS roles

**DOI:** 10.1186/s12909-023-04004-x

**Published:** 2023-02-15

**Authors:** Farhan Mahmood, Julianah O. Oguntala, Claudine Henoud, Libny Lahelle Pierre-Louis, Asli Fuad, Ike Okafor

**Affiliations:** 1 Faculty of Medicine, University of Ottawa, Ottawa, ON Canada; 2 Temerty Faculty of Medicine, University of Toronto, Toronto, ON Canada; 3 Office of Vice Dean, Strategy and Operations, Temerty Faculty of Medicine, University of Toronto, Naylor Building, Rm 311 6 Queen’s Park Cres W, Toronto, ON M5S 3H2 Canada

**Keywords:** Medical education, Admissions, Medical students, Diversity, Medical school application, CASPER, Standardized test, Medical innovation, Leadership, Mentorship, CASPER snapshot, CanMEDS

## Abstract

**Background:**

Underrepresented Minorities in Medicine (URMMs) may face financial and social limitations when matriculating into medical schools. Performance on situational judgment tests such as Computer-based Assessment for Sampling Personal Characteristics (CASPER) can be enhanced by coaching and mentorship. The CASPER Preparation Program (CPP) coaches URMMs to prepare for the CASPER test. During the coronavirus 2019 pandemic (COVID-19), CPP implemented novel curricula on the CASPER Snapshot and CanMEDS roles.

**Methods:**

Pre and post-program questionnaires were completed by the students, which assessed their: 1) confidence in understanding the CanMEDS roles, and 2) perceived confidence in performing well and their familiarity and preparedness with the CASPER Snapshot. With a second post-program questionnaire, participants’ scores on the CASPER test as well as medical school application outcome were also assessed.

**Results:**

Participants reported a significant increase in the URMMs’ knowledge, self-perceived competency to complete the CASPER Snapshot, and their anxiety significantly decreased. The level of confidence in understanding CanMEDS roles for a career in healthcare increased as well. The majority (91%) agreed that the feedback received from tutors was adequate and the virtual component of the program was beneficial during COVID-19. 51% of students scored in the highest quartile on the CASPER test and 35% received an offer of admission from CASPER-requiring medical schools.

**Conclusion:**

Pathway coaching programs have the potential to increase confidence and familiarity amongst URMMs for the CASPER tests and CanMEDS roles. Similar programs should be developed with the aim to increase the chances of URMMs matriculating into medical schools.

## Introduction

The diverse Canadian population should be reflected amongst Canadian medical students and physicians to ensure patients are provided socially and culturally competent care [1–3]. Canadian medical schools lack representation of several underrepresented minorities in medicine (URMMs) including but not limited to Black and Indigenous students and students with low socioeconomic status backgrounds (SES) [4–6].

The Canadian medical school application involves the evaluation of applicants' academic and non-academic parameters including references and autobiographical sketches. Some medical schools also require situational judgement tests (SJTs) such as the Computer-based Assessment for Sampling Personal Characteristics (CASPER) [7–10]. A competency-based framework approach called the Canadian Medical Education Directives for Specialists (CanMEDS) includes six key roles defining the Medical Expert: Communicator, Collaborator, Health Advocate, Manager, Scholar and Professional [11–13]. Admission offices in Canadian medical schools, such as the Temerty Faculty of Medicine at the University of Toronto, are also increasingly requiring their matriculants to embody the CanMEDS competencies [11–13].

However, URMMs face various admission inequities contributing to the lack of diversity seen in medical education. They receive lower scores on traditional tests of scholastic ability, are more likely to lack the social capital to navigate a field like medicine, and experience financial barriers [9, 14]. However, differences between mean scores amongst different demographics are narrower for SJTs compared to cognitive tools like the Medical College Admissions Test (MCAT) and grade-point averages (GPA) [9]. Specifically, the effect size difference for African American applicants compared to white applicants on the MCAT and GPA was 1.43 and 0.98, respectively [9]. This is in contrast to a CASPER effect size difference of 0.60, which was statistically significant [9]. Hence, the CASPER test, an SJT, can potentially reduce demographic disparities in the admissions process [9]. Furthermore, although performance on SJTs have been shown to improve with coaching [15, 16], many URMMs lack the funds to enrol in for-profit coaching programs to improve their scores on CASPER [17].

Due to COVID-19, a new component of CASPER was introduced, called the CASPER Snapshot [8], allowing admissions committees to learn more about their applicants in a virtual setting. Applicants are required to answer three questions about their motivation to pursue medicine, future goals and passions, in a ten minute live recorded video [8, 18].

This year, the CASPER Preparation Program (CPP), described in detail in the methods section, implemented novel curricula on the CASPER Snapshot and the CanMEDS. In previous iterations of the program, learners raised questions on what the CanMEDS roles are and how they are used in evaluating medical school applicants. Therefore, curricula on the CanMEDS roles were introduced to increase the awareness of CPP learners of the competencies they are to represent in their applications and they are to embody as medical school graduates.

This study aims to assess the efficacy of CPP’s novel curricula, further elucidating how pathway programs can adapt to the novel components of the medical school admissions process to mitigate barriers that URMMs face during the application process.

## Methods

**Fig. 1:**
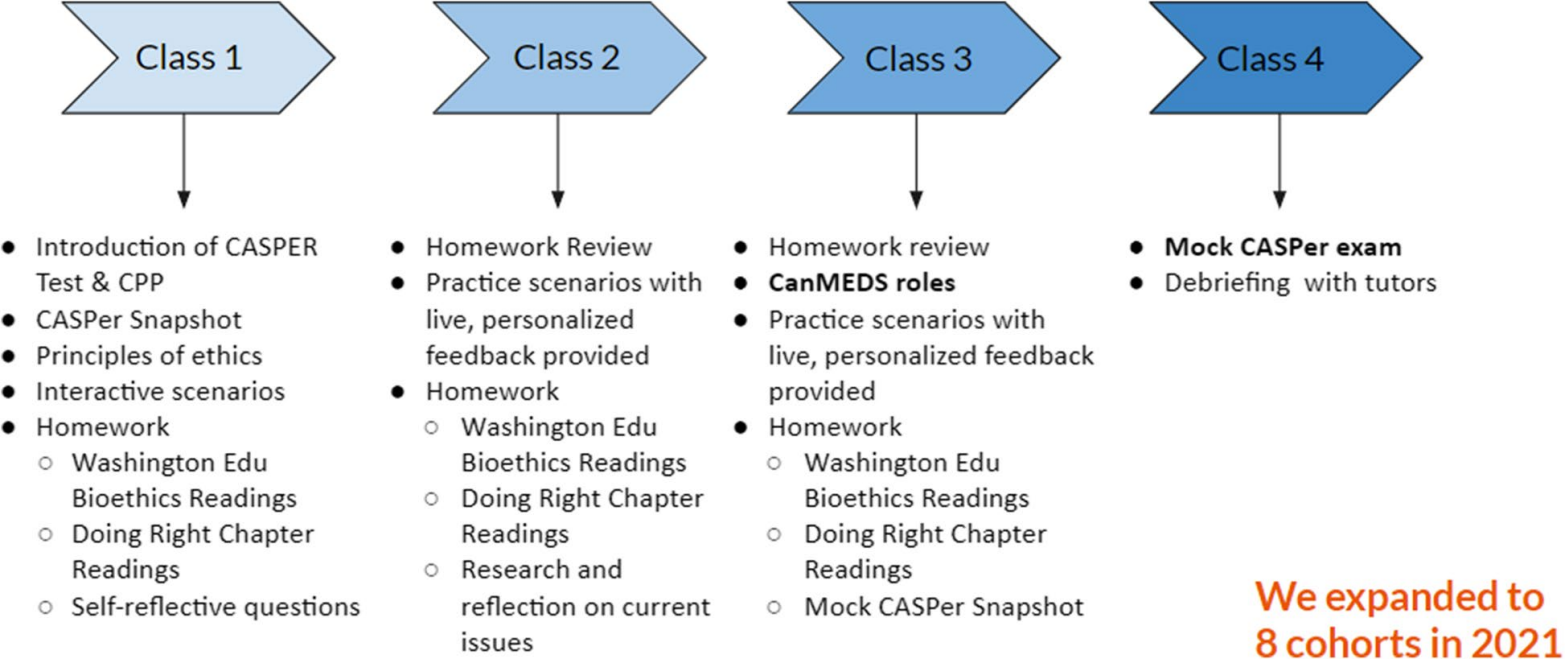
A breakdown of the sessions of the CASPER Preparation Program (CPP)

CPP was launched in 2019 by medical students from the University of Ottawa in collaboration with the Community of Support (COS) program at the University of Toronto [19]. The free program has run for 3 cycles virtually in 2019, 2020, and 2021, independently of Altus Assessments, the creators and administrators of the CASPER test [8]. Shipeolu et al. [20] previously described the detailed structure and components of CPP coaching URMMs for the CASPER test. Figure 1 also provides a brief overview of the 4 sessions. In 2019, there was 1 cohort consisting of 35 students and in 2020 there were 3 cohorts with 140 students. In 2021, this program expanded to 8 cohorts (25–45 students per cohort) with a total of 333 URMMs. URMMS were defined as, but not limited to, students self-identifying as Black, Indigenous, and Peoples of Colours, students with disabilities and students from low SES backgrounds.

The CPP course consists of four 2 h sessions with 15 min of office hours following each session. The CPP course content development was guided by the Altus Assessment description of the program with the content of each of the four sessions described in Fig. 1. During the sessions, 3 medical students, known as tutors, discussed the CASPER test structure, ethical decision-making, the CASPER Snapshot, the CanMEDS roles, reviewed ethics-based cases, and provided direct and personalized feedback on the homework and mock exam/question responses. Office hours provided an opportunity for learners to ask clarification questions of the tutors.We had 13 unique tutors teaching the 8 cohorts and a majority of the tutors are also identified as URMMs. All tutors have previously written the CASPER test and received a virtual training session before the program began on the course material. The course content was exactly the same between cohorts.

This year, CPP incorporated lessons about the CanMEDS roles [19], the CASPER Snapshot [8], and developed a CASPER Snapshot homework assignment with personalized feedback. This innovation is the first of its kind, as CASPER Snapshot was recently developed in 2020 by Altus Assessment, and is required by several medical schools as part of the admissions process [8]. CPP participants were asked to submit videos simulating the CASPER Snapshot, after which they received feedback from tutors about their communication styles, quality of responses, and areas for improvement.

Participants from the June and July cohorts anonymously and voluntarily completed surveys before (pre-course) and after (post-course) the CPP sessions. The surveys assessed parameters including course structure, self-perceived confidence in understanding CanMEDS roles, and self-perceived confidence and competence to perform well on the CASPER Snapshot using 5-point Likert scales. The questionnaires were modeled after questionnaires described by Gostelow et al. [21] and Shipeolu et al. [20]. This program evaluation was exempted by the University of Ottawa’s Research Ethics Board.

In 2022, the 2021 CPP participants were invited to complete a second post-course survey that assessed their quartile ranking on the CASPER test as well as with their medical school applications.

Data was presented as the mean and standard deviation of responses on the 5-point Likert scale such as 1 (strongly disagree), 3 (neutral), and 5 (strongly agree). The student’s t-test was used to compare the aggregated pre-and post-course questionnaires (*P*-value < 0.05 was considered significant).

## Results

A total of 126 and 60 URMMs completed the pre-and post-course questionnaires, respectively (response rates 84.6%, 40.3%, respectively). Respondents who indicated they did not complete the pre-course questionnaire in the post-course questionnaire were eliminated from the data analyses.

**Fig. 2:**
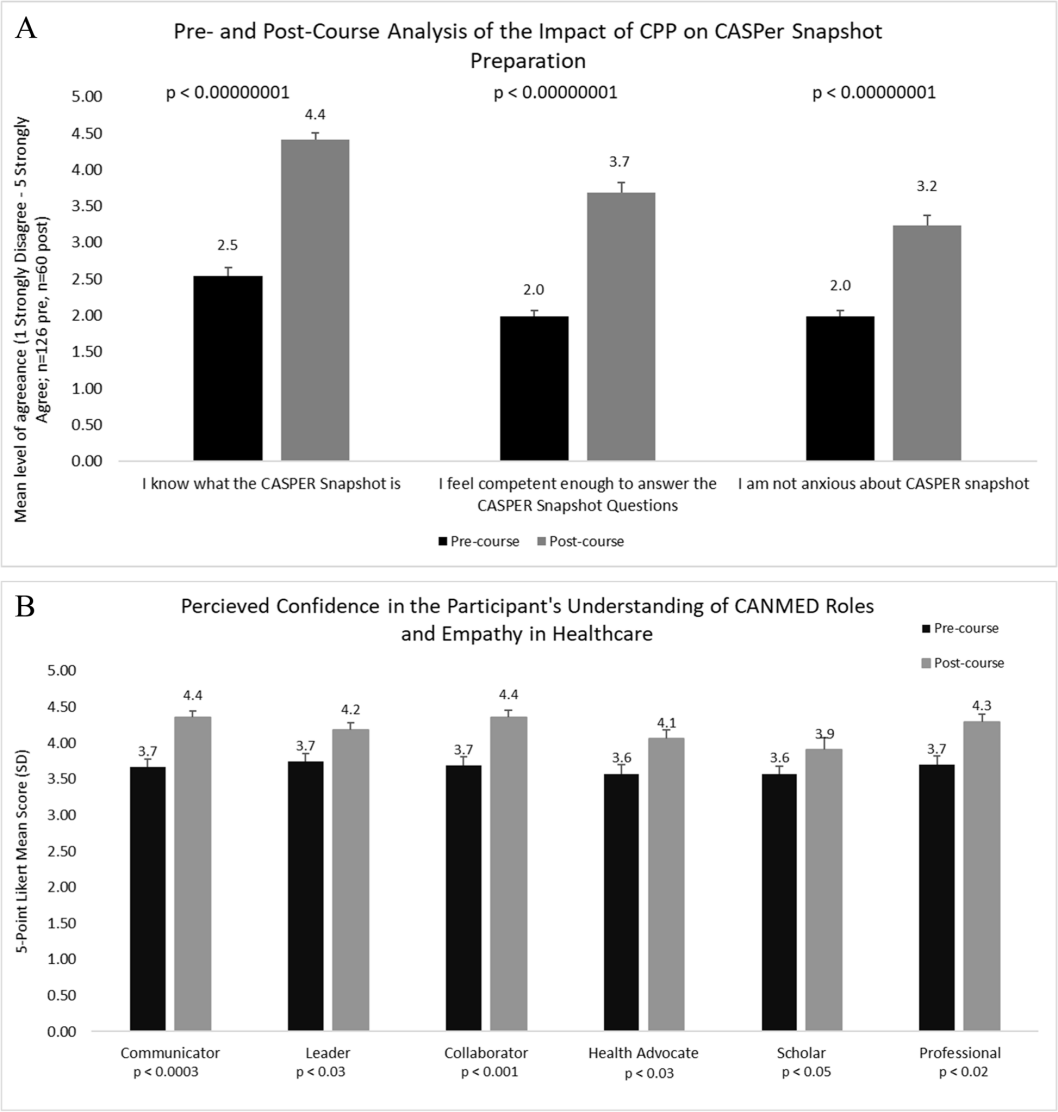
**A** Pre- and Post-Course Analysis of the Impact of CPP on CASPER Snapshot Preparation. **B** Perceived Confidence in the Participant's Understanding of CanMEDS Roles in Healthcare

Compared to prior to starting CPP, there was a significant increase in knowledge about the CASPER Snapshot after completing CPP (2.54 ± 1.37 versus 4.42 ± 0.65), and the mean level of self-perceived competence to answer CASPER Snapshot questions increased significantly after CPP as well (1.98 ± 0.89 versus 3.682 ± 1.05), all *p* < 0.0000001. The applicants were significantly less anxious about the CASPER Snapshot (*p* < 0.0000001) (Fig. 2).

The majority (60%) of URMMs agreed or strongly agreed that the Mock CASPER Snapshot exercise homework and personalized feedback were very helpful. 35% of the applicants were neutral, and 5% of the applicants disagreed or strongly disagreed.

The level of confidence in understanding the roles of CanMEDS for a career in healthcare increased after completing the July cohorts (*n* = 77 for pre-course and *n* = 34 for post-course questionnaires). Data from the two July cohorts were grouped together. This outcome was not assessed in the June cohort. There was a significant increase in understanding the roles of “communicator” (*p* < 0.0003), “leader” (*p* < 0.03), “collaborator” (*p* < 0.001), “health advocate” (*p* < 0.03), and “professional” (*p* < 0.02) roles (Fig. 2). The increase in understanding for the “scholar” role was insignificant (*p* < 0.09).

Most of the URMMs felt that the course load in the in-class sessions and homework was “just enough” (77 and 80%, respectively). The overall assessment of the course was mostly “excellent” or “very good” (88%), and 91% of URMMs agreed that the tutors’ feedback was adequate and helpful (Table 1). CPP participants found the strong points of the course to include the personalized and live feedback received at various points of the course and the multiple perspectives provided by the 3 tutors in each class. The mock exam administered at the end of the class with feedback provided, the small class sizes and utilization of breakout rooms were received. Participants further positively commented on the incorporation of CASPER Snapshot and the CanMEDS roles in the courses.

**Table 1:** Summary of feedback from underrepresented medical school applicants about the CASPER Preparation Program

*N* = 60	Strongly Agree (%)	Agree (%)	Neutral (%)	Disagree (%)	Strongly Disagree (%)
The feedback received was adequate and helpful	43	48	2	7	0
	Excellent (%)	Very Good (%)	Good (%)		
What is your overall assessment of CPP?	40	48	12		
	Just Enough (%)	Too Few (%)	Too Many (%)		
How would you describe the course load of CPP classes?	77	23	0		
How would you describe the course load of CPP homework?	80	12	8		
**Major themes/comments & General Recommendations for Medical Innovation Programs**					
What do you think were the strong points of this course?	○ Personalized and live feedback in-class, for homework, mock exams ○ CASPER mock exam and feedback ○ In-class examples for ethics-based questions ○ Doing Right reading assignments and discussion ○ The presence of multiple tutors providing their perspectives ○ Incorporation of CanMEDS roles in the curriculum ○ Incorporation of the CASPER Snapshot in the curriculum ○ The positive learning environment created by the course tutors ○ The small class sizes and break out rooms/group exercises				
What could have been improved about the course?	○ Developing a question bank ○ Incorporate more cases from Doing Right readings ○ More sessions (more than 4 sessions per group) ○ Longer sessions (current sessions are 2 h) ○ More time dedicated to the breakout room sessions ○ More time dedicated to in-class practice questions ○ Content on how to identify ethics-based questions ○ Content on how to apply CanMEDS roles into the responses ○ Detailing a specific structure on how to answer ethics-based questions ○ Providing tutor responses for all the practice questions ○ Providing feedback to every student's response during in-class practice sessions ○ Incorporating the in-class scenarios into the homework and providing direct feedback ○ Providing feedback for homework and mock exams sooner ○ Reducing the number of assigned readings from Doing Right ○ Longer access to the electronic book “Doing Right” ○ Offering more mock exams to be completed after the program ○ Tutors should manage time better during the sessions (e.g., starting and ending the class on time)				

In terms of improvement, CPP participants suggested having more than 4 sessions per course, allocating more time for the breakout rooms as well as providing sample tutor responses for the practice questions. Table 1 further summarizes the strength and weaknesses of CPP and recommendations for other pathway medical innovation programs to consider.

43 2021 CPP participants completed the second post-course survey released in 2022. 91% of these students wrote the CASPER test after participating in CPP. 51% of students scored in the highest quartile with another 14% scoring in the second highest quartile. 88% of students went ahead of apply to medical school with 84% applying to at least 1 CASPER-requiring school. 70% of students received at least 1 interview invitation, 56% received an interview to at least one CASPER-requiring medical school. 58% of students received an offer of admission to at least one medical school and 35% received an offer from at least one CASPER-requiring medical school.

## Discussion

CPP targets URMMs to help them overcome financial barriers associated with preparatory CASPER courses, ultimately aiming to increase diversity and representation in healthcare. Increasing diversity in healthcare is necessary as healthcare representation of underrepresented groups allows for better patient care, access, and outcomes [1, 2]. Addressing the lack of diversity in healthcare requires changes to be made during the admission process [4, 6, 7, 9].

The launch of CASPER Snapshot may have left students with many uncertainties especially during the pandemic. Additionally, the CanMEDS roles parallel the qualities evaluated on the CASPER test as well in medical schools applications. CPP implemented novel coaching materials and due to the interview-style structure of Snapshot, tutors who had not completed it were still able to provide feedback from their interview experiences. The video component of CASPER Snapshot currently replicates the online medical school interviews that are also being held due to the COVID-19 pandemic. A majority of students agreed that the mock CASPER snapshot was helpful and there was a significant increase in self-perceived competence to answer the questions. Introducing the CanMEDS roles to the course increased the confidence of CPP participants on how they apply to a career in healthcare. Due to the way the question was presented, it is difficult to delineate the percentage of students who found the course adequate but not helpful and vice versa.

The CPP participants also provided areas of strengths for the course as well as improvement. The framework of providing personalized feedback in-class, on homework and on a mock CASPER test was well received. However, participants also wanted more time allocated for smaller group discussions and the offering of additional mock tests after the completion of the program. These are improvements that will be considered in future iterations of CPP.

A significant proportion of CPP students progressed to write the CASPER test, apply to medical school and receive offers of admission to both CASPER requiring and non-requiring medical school. These findings support the success of CPP and the positive impact it has on the journey of participants.

Beyond the support CPP provides in terms of CASPER Snapshot and the CanMEDS roles, by being a free program, CPP also provides some financial relief for students. Additionally, as the course is taught by medical students, CPP provides access to mentorship for its learners and allows for safe learning experiences. Many of our learners will be the first to attend medical school in their families, therefore CPP allows them to interact with peers of similar lived experiences, increasing their social network in medicine.

Since 2019, CPP has expanded in capacity, collaborating with other medical schools and students across Ontario including McMaster and Dalhousie Universities [20]. The program’s long-term goals include providing cohorts in French, recruiting more URMMs nationally, and preparing additional free resource materials for students. The leaders of CPP also hope to encourage and inspire medical students and Faculties of Medicine to be socially accountable. Programs similar to CPP need to be developed at and supported by schools that require the CASPER test to further increase capacity and reach more URMMs. Similar to the supports CPP has received from McMaster and Dalhousie, other medical schools need to step-up to support coaching programs, which is in line with calls to action to increase representation in medical schools across the country.

CPP has received financial support from the University of Toronto Temerty Faculty of Medicine, Dalhousie Faculty of Medicine and the University of Ottawa Faculty of Medicine Aesculapian Society. Further financial and in-kind support will be needed from other CASPER-requiring medical schools for future iterations of CPP to reach more students and continue to improve diversity in the medical student and physician body.

### Limitations

Participants' self-perceived competence was subjective and may not correlate with success on the CASPER test or matriculation. We do not have access to the students’ official CASPER scores nor any official information pertaining to their interview invitations and admissions. We instead relied on the information provided by students and not all students responded in the second post-program questionnaire. The pre-and post-course survey responses concerning the CanMEDS roles were only available for the July cohort resulting in a smaller sample size. The program’s impact on the target population may be limited because we did not verify the participants’ self-identification. Finally, as each cohort was taught by different tutors, it is possible that the participants’ experiences varied between cohorts. We attempted to minimize this difference by utilizing the same teaching materials across the entire course, having a small number of tutors deliver the course and providing the same training to the tutors.

## Conclusion

2021 CPP outcomes were described by comparing the pre-and post-course surveys assessing the students’ self-perceived confidence, competence, and knowledge of the CanMEDS roles and of the CASPER Snapshot. Despite its recent introduction, most students felt more competent and more confident in taking the CASPER Snapshot following the CPP, which included a mock CASPER exam. Additionally, personalized and live feedback provided by tutors was beneficial to participants. Moving forward, we hope to further investigate CPP’s efficiency in terms of admission to the medical program. We also hope to expand the program and increase the socioeconomic and cultural diversity in Canadian medical schools which in turn may lead to a more diverse physician workforce, more capable of serving underrepresented populations.

## Data Availability

The datasets used and/or analysed during the current study available from the corresponding author on reasonable request.
